# Insights Into the Origin and Deformation Style of the Continental Moho: A Case‐Study From the Western Alps (Italy)

**DOI:** 10.1029/2020JB021319

**Published:** 2021-06-23

**Authors:** Simone Salimbeni, Nicola Piana Agostinetti, Silvia Pondrelli, CIFALPS Working Group

**Affiliations:** ^1^ Istituto Nazionale di Geofisica e Vulcanologia Bologna Italy; ^2^ Department of Earth and Environmental Sciences University of Milano Bicocca Milan Italy; ^3^ Department of Geodynamics and Sedimentology University of Vienna Vienna Austria; ^4^ All CIFALPS Working Group details are presented in the acknowledgments section

**Keywords:** receiver functions, harmonics decomposition, Continental Moho, Ivrea body, seismic anisotropy

## Abstract

Several hypotheses on the origin of the continental Moho are still debated and multiple mechanisms may contribute to its formation. Here, we present quantitative estimation of the seismic properties and anisotropy of the crust‐mantle transition in the Western Alps where an example of newly formed (proto)‐continental Moho is unusually shallow. We make use of teleseismic P‐to‐S converted‐waves recorded by stations deployed on top of the Ivrea Body (IB), a volume of possibly serpentinized mantle peridotite below exhumed (ultra‐)high pressure crustal rocks. The IB has been mapped by gravity, magnetic, active and passive seismic surveys suggesting an extremely shallow Moho. We demonstrate that the P‐to‐S converted waves propagating through this region display coupled features: (a) they record expected presence of strong seismic velocity contrast at shallow depth as due to the lower crustal and upper mantle transition; (b) they are decomposed due to anisotropic properties of rocks involved. The proto‐continental Moho is recognized as an increase in S‐wave velocity (∼0.4–1 km/s) at shallow depths of 5–10 km. The presence of anisotropy within the IB and overlying crustal rocks is evidenced by back‐azimuthal dependence of the amplitude of P‐to‐S phases. The strength of anisotropy is ∼−14% on average pointing out the presence of metamorphosed/hydrated material (e.g., serpentinite) below the Moho. Anisotropic directions are consistent across Moho in both crust and upper mantle. The similarity of the anisotropy parameters between crust and upper mantle suggests they have been shaped by the same deformation event.

## Key Points

- Receiver Functions and harmonics decomposition
- Continental Moho and Ivrea Body
- Seismic anisotropy

## 1 Introduction

In contrast with the crust‐mantle boundary beneath oceans, whose genesis and physical properties have been studied long ago, the seismic properties, nature and origin of the continental Moho have long been debated (Eaton, 2006; O'Reilly & Griffin, 2013). The knowledge of seismic properties of lower‐crustal and upper‐mantle rocks including their anisotropic fabrics is fundamental in modeling realistic geodynamic scenarios of, for instance orogen build‐up, crust‐mantle decoupling and the dynamics of lithospheric fault zones (Carbonell et al., 2013; Jull & Keleman, 2001; Platt & Behr, 2011). The nature of the continental Moho has been previously studied worldwide using geophysical investigations for well‐defined tectonic regions like cratonic shields (e.g., Canada, Jones & Ferguson, 2001), collision zones (e.g., Himalaya, Nábělek et al., 2009) or extensional settings (e.g., African rift, Long et al., 1973). Due to the depth of the target rock volume (20–70 km), such investigations have generally been hampered by low vertical resolution and have not been able to shed full light on many open questions including the sharpness of the velocity change at the continental Moho (gradational boundary or sharp interface) or the anisotropic properties of crustal and upper‐mantle rocks close to Moho boundary.

**Figure 1:**
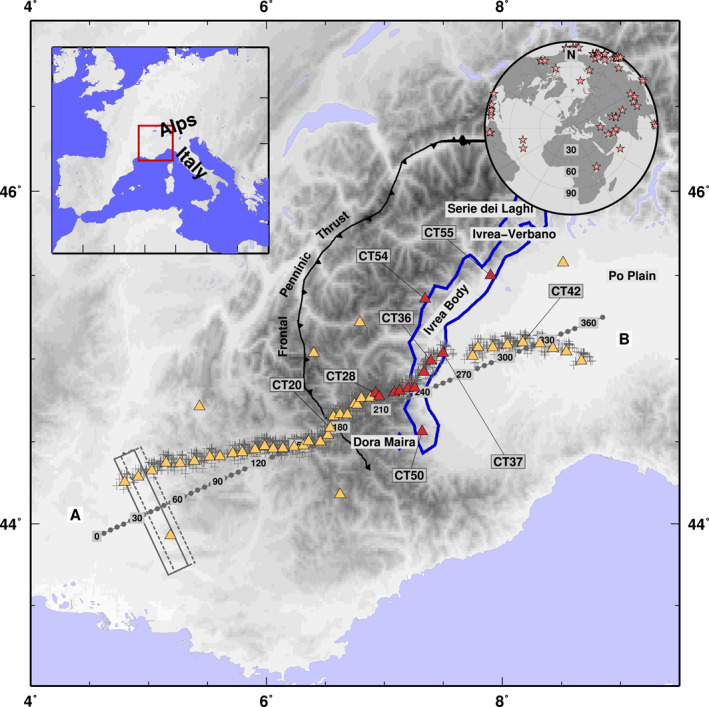
Topographic map of the study region. Yellow and red triangles are the CIFALPS seismic stations, with red triangles showing stations located in the Ivrea positive gravity anomaly (blue continuous line, Bonvalot et al., 2012). Crosses are piercing points for each earthquake analyzed at each station. Profile A and B is used to project results in the next figures. Width used for the profile projection and example of dimension of overlapping‐window used in the binned procedure are indicated by continuous and dashed gray boxes. Top Left: Geographical framework of the Western Alps region. Top right: map of earthquakes used to obtain receiver functions (RFs).

The Ivrea Body (hereinafter IB; Figure 1) has an important role in the reconstruction of the structure and evolution of the Western Alps. It is related to a major high gravity and high seismic velocity anomaly, recognized since the early 1960s (Berckhemer, 1968; Closs & Labrouste, 1963; Scarponi et al., 2020 and references therein). Just a small portion of it, named the Ivrea‐Verbano zone (Northwest Italy, Figure 1) is exposed at the surface and represents the world's best outcrop of lower crustal continental rocks (Fountain, 1976). The discovery of refracted waves at 7.4 km/s at 10 km depth and modeling of the positive Bouguer anomaly led to consider the IB as a sliver of Adriatic mantle rocks at a very shallow depth in the alpine crust (Closs & Labrouste, 1963). It has been brought close to the surface during Tethyan rifting (Manatschal & Bernoulli, 1999) and then involved in the multi‐stages evolution of the Western Alps arc during the European‐Adriatic plate convergence (Schmid et al., 2017), that took place through the subduction of the European under the Adriatic plate (Zhao et al., 2015, 2016) up to a continental collision. The IB had a crucial role in the separation of the type of tectonic structure and crustal shortening along the Alpine chain as recent numerical thermomechanical modeling demonstrated (Liao et al., 2018). The transition zone between the IB and the overlying lower crustal rocks of the Ivrea‐Verbano zone to the North and the (ultra)high‐pressure (U‐)HP metamorphic rocks of the Dora Maira massif to the South is thus one of the shallowest continental Mohos on Earth. This shallow Moho will be one of the objectives of the DIVE project (Drilling the Ivrea‐Verbano zonE, https://www.icdp-online.org/projects/world/europe/ivrea-italy/details/) that was recently integrated in the roadmap of the International Continental scientific Drilling Program. The DIVE project will address fundamental questions about the origin of the continental crust, its formation and evolution (Pistone et al., 2017). However, while the characterization of the northern part of the IB benefits of studies on lower‐crustal rocks of the Ivrea‐Verbano zone (i.e., Pistone et al., 2020; Scarponi et al., 2020), the extension and definition of the IB in its central and southern parts is not straightforward, due to the difficulties and challenges in mapping subsurface rock volumes (Fountain, 1976). A recent modeling of a dense gravity data set in the Ivrea‐Verbano zone has shown that the Moho on top of the IB may be as shallow as 1–2 km below sea level (Scarponi et al., 2020). The IB is therefore a perfect target for investigating the sharpness of the seismic velocity change at the continental Moho and the anisotropic properties of lower‐crustal and upper‐mantle rocks.

Receiver Functions (RFs) is a widely used tool for reconstructing crustal and upper‐mantle seismic structures, based on the analysis of P‐to‐S converted phases in teleseismic P‐wave coda (Langston, 1979; Vinnik & Montagner, 1996). The vertical resolution of RF within the shallow crust can be better than 1 km (Piana Agostinetti & Malinverno, 2018) and the analysis of RF datasets can be used to probe seismically anisotropic bodies at depth (Bianchi et al., 2010). Indeed, RF data sets have been analyzed for estimating anisotropic parameters of, for example, subducted crust (Piana Agostinetti & Miller, 2014), flow paths in volcanic districts (Bianchi et al., 2008), and fracture networks in geothermal fields (Piana Agostinetti et al., 2017).

Since the early 1960s, the IB has been mapped at depth using passive seismic tomography and Receiver Function analysis (Diehl et al., 2009; Paul et al., 2001; Scafidi et al., 2006; Solarino et al., 2018; Zhao et al., 2015 and references therein), with a robust correlation with the impressive positive Ivrea gravity anomaly (Kissling, 1984). In particular, the RF analysis of Zhao et al. (2015) identified a positive P‐to‐S (Ps) pulse, generated at 10–15 km of depth beneath the Dora Maira massif and the westernmost Po Plain. They interpreted this interface as the top of the Ivrea Body rocks, that is, part of the Adriatic mantle at the upper crustal depth as already suggested by Nicolas et al. (1990). The bottom of that body is located around 30 km. Zhao et al. (2015) proposed for that body a velocity and density model compatible with the presence of peridotitic composition rocks with different degrees of serpentinization. These results were later strengthened by local (Solarino et al., 2018), full‐waveform (Beller et al., 2018) and ambient noise tomographies (Lu et al., 2018; Zhao et al., 2020).

In this study we aim to investigate the seismic properties and the presence of anisotropy across a newly formed (proto‐) continental Moho, that is, continental Moho which we assume will undergo to additional geophysical processes in the near‐future (in a geological time‐scale). The presence of the IB offers a unique opportunity to take a picture in great detail of a process that is usually buried at un‐reachable depth (i.e., where resolution of standard seismic techniques is poor). We exploit the information contained in the data recorded by the CIFALPS temporary seismic experiment (Zhao et al., 2016 and Malusà et al., 2021 for an extensive review). We apply standard RF analysis to the relevant stations of the CIFALPS network to invert for both the isotropic and anisotropic structures of the crust and upper mantle. The estimated parameters allow to define a conceptual model of the origin of this shallow proto‐continental Moho and to provide new insights into its deformation style.

## 2 Data and Methods

**Figure 2:**
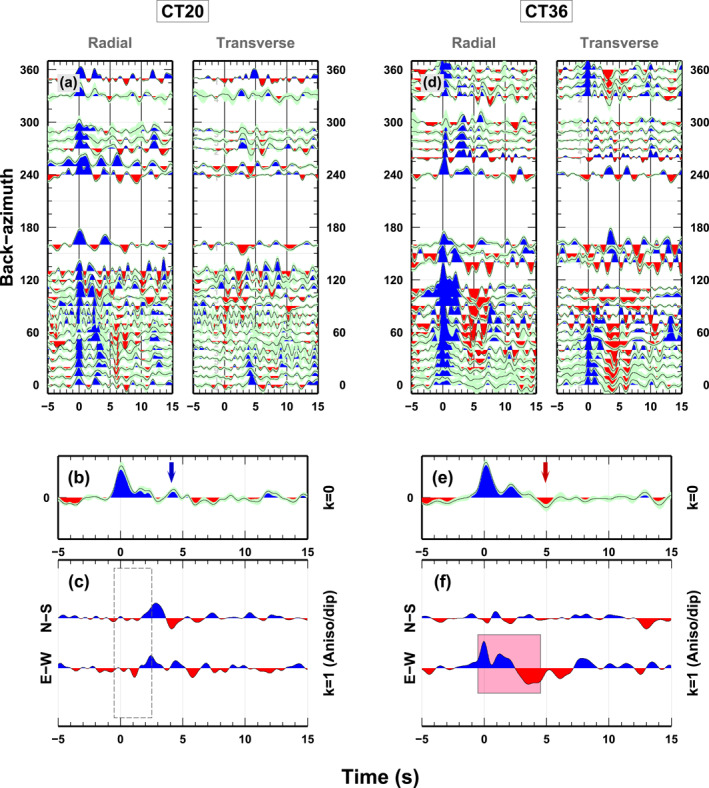
Example of receiver functions (RF) (panels a and d) and first and second harmonics (panels b, c, e and f) for stations CT20 and CT36, stations located along the Frontal Penninic Thrust (FPT) and on top of the Ivrea body (IB), respectively. In panels (a) and (d), binned RF data as a function of the back‐azimuth of the incoming P‐wave are drawn, both for Radial (left) and Transverse (right) components. Colors represent positive (blue) and negative (red) amplitudes of the P‐to‐S converted waves. The green‐ish area along each single bin shows the standard deviation associated to each point of the bin. Blue arrow in panel (b) highlights the positive pulse that characterizes CT20 isotropic part of the signal. The red arrow in panel (e) indicates the uncommon negative pulse in the isotropic part of the signal of CT36. In panel (f), two opposite and energetic pulses are present on *k* = 1 harmonic (pink square on panel f) in the first 4 s 0–4 s, with most of the energy located in the E‐W component. This feature is absent at station CT20, dashed box in panel (c).

The 56 stations of the temporary CIFALPS seismic experiment (07‐2012 to 09‐2013; Zhao et al., 2016; https://doi.org/10.15778/RESIF.YP2012; A‐B transect in Figure 1) provide records of a fair number of *Mw* ≥ 5.0 teleseismic events in the 30°–100° epicentral distance range with sufficient back‐azimuthal coverage for our analysis. From each 3‐component recording of a teleseismic wave, we compute the radial R‐RF and transverse T‐RF following the approach described in Di Bona (1998). We obtain between 154 and 398 couples of RF for each station and we visually select the RF with a high S/N ratio, trying to extend our back‐azimuthal coverage as much as possible. Our final data set includes 2227 couples of high quality RF, with a minimum of 12 (station CT02) and a maximum of 75 (station CT16) for each station. RF are computed with a frequency‐deconvolution method (Di Bona, 1998). We make use of a Gaussian filter with *a* = 2, that is, low‐passing the signal to ∼1 Hz, thus the vertical resolution is about 1 km in the upper crust and 2–3 km in the deeper part of our models (Piana Agostinetti & Malinverno, 2018). Such methodology estimates the variance associated with each single couple of RF, allowing us to weight the RF in the following procedure. For visual inspection of the data at each station, we compute a binned RF data set, where computed RF from events coming from the same seismogenic area are stacked together (i.e., all events in a 10° × 20° back‐azimuth *x* distance area are stacked together using the inverse of their estimated variance as weights). As an example of the obtained information, we present in Figure 2 the binned RF data set for two stations deployed in different tectonic settings: CT20 in the external Alps close to the Frontal Penninic Thrust (FPT hereinafter, Figure 1), and CT36 above the IB. In panels (a) and (d), we display the binned RF data as a function of back‐azimuth of the incoming P‐wave for the radial and the transverse components. The back‐azimuthal coverage is incomplete but sufficient for harmonic analysis.

Radial and transverse RF data sets can be combined to extract their angular harmonics as a function of back‐azimuth (Bianchi et al., 2010). The first (*k* = 0) harmonic contains information about the isotropic structure beneath a seismic station, that is, the bulk seismic velocity variations. Conversely, the amplitude of P‐to‐S converted waves generated by seismic waves traversing anisotropic rocks displays a characteristic pattern with a 360° periodicity recorded on the second (*k* = 1) harmonic (Park & Levin, 2016). Separating these two harmonics helps in detecting anisotropic bodies at depth and in interpreting a RF data set (Licciardi & Piana Agostinetti, 2016). Uncertainties on harmonic coefficients are computed through a bootstrap procedure (Bianchi et al., 2010). In Figures 2b, 2c, 2e and 2f, we show examples of the first and second harmonics of two RF data sets for stations CT20 and CT36. In panels (b) and (e), the amplitude of the first harmonic is shown, with each pulse representing a seismic velocity jump at depth. As a rule of thumb, blue (red) pulses represent seismic velocity jump where velocity increases (decreases) with depth. In panels (c) and (f), we show the second *k* = 1 harmonic. In this case, energy is decomposed along two orthogonal axes (North‐South and East‐West). The arrival times of the most energetic pulses on the *k* = 1 harmonic indicate at which depth seismic anisotropy is confined.

To quantify the physical properties of rocks composing the IB, we solve the geophysical inverse problem of reconstructing anisotropic one‐dimensional S‐wave velocity profiles at each station. We apply the inversion workflow to the RF data sets of the 12 seismic stations deployed on top (or close to) the positive gravity anomaly (red triangles in Figure 1) for which RF data set displays the key‐features described above, plus 2 stations outside the IB area, for comparison. We consider a simplified anisotropic model of hexagonal anisotropy, which is widely representative of the anisotropic conditions at depth (Becker et al., 2006). Hexagonal anisotropy can be represented using three parameters, that is two angles (trend and plunge) and the intensity (same intensity assumed for P‐ and S‐wave anisotropy). Forward predictions are computed using RAYSUM code (Frederiksen & Bostock, 2000). We adopt a long‐standing algorithm (Neighborhood Algorithm, NA, Sambridge, 1999) for exploring a parameter space composed of a stack of horizontal isotropic and/or anisotropic layers, where the axis of symmetry is freely oriented in the three‐dimensional space (Table S1). The exact definition of the parameter space must be driven by key features observed in the RF data set described in Section 3.3. For each station, we sample 21,000 models. The first Ns1 = 1,000 models are randomly sampled in the parameters space. Then, the Nc = 5 Voronoi cells, belonging to the five best‐fit models, are resampled 8 times each, for a total of Ns2 = 40 new models. This operation is re‐iterated Niter = 500 times. The full ensemble of 21,000 sampled models allows us to estimate uncertainties on the investigated parameter, rather than just defining the best‐fit model. Here we select a “best‐fit family” including the models with a fit smaller than 10% the best‐fit model. Quantitative estimates of the parameters are retrieved from this family (e.g., best‐fit value, average value, min/max value and standard deviation).

## 3 Results

### 3.1 Back‐Azimuthal Harmonics of Single Station RF Data Set

We compute the receiver function data set and its harmonic decomposition independently for each station belonging to the CIFALPS temporary experiment. The single station RF data sets evidence the high degree of heterogeneities in the crustal and upper mantle structures. In particular, it shows strong differences between stations located inside and outside the IB area. In the stable part of the European plate, where for instance CT20 is located (Figure 2a), the radial RF data set displays a rather simple pattern in the first two seconds, with respect to, for example, CT36 (Figure 2d). The RF data set for CT20 displays a clear direct‐P arrival and a positive pulse between 3 and 5 s on the radial component while a negative pulse is well visible at ∼6 s especially in the 0°–120° back‐azimuthal range where the teleseismic coverage is better. In the transverse component, no arrivals are present in the same time‐window except for a positive and not continuous pulse at 6 s (panel a in Figure S1). These features are almost continuous between neighboring stations. In contrast, stations located above the IB (i.e., CT36 in Figure 2d) display a direct‐P pulse with more complex variations with back‐azimuth. More interestingly, a broad negative pulse is observed in the 3–6 s time interval on the radial components, coupled with a large amplitude arrival on the transverse components, with a clear change in polarity around 180° and 300° in back‐azimuth (Figure 2d). The differences between RF data of CT20 and CT36 confirm that the structures at 10–50 km depth under these 2 stations are very different.

We now focus on the decomposition of the RF in their first and second angular harmonics. Panels (b–c) and (e‐f) in Figure 2 emphasize again differences in the crustal structure and in the anisotropic properties between CT20 and CT36. CT20 and other stations located outside the IB region (see also Figures S1 and S2) show no energetic pulses in the 0–3 s time‐window of the *k* = 1 harmonics. This allows to exclude the presence of any anisotropic rock at shallow depth (dashed box in Figure 2c). A clear positive arrival at ∼4 s on the *k* = 0 harmonic may indicate the Ps converted phase at a 30‐to‐40 km depth Moho. Conversely, the *k* = 1 harmonic at station CT36 (and others located above the IB) displays the two most energetic pulses between 0‐5 s (pink square in Figure 2f) pointing out the presence of highly anisotropic rocks in the entire crust. Most of the energy is located in the E‐W component of the *k* = 1 harmonic, along which the trend of the symmetry axis of the material is possibly oriented. On the *k* = 0 harmonic, a large amplitude positive pulse at ∼2 s testifies the presence of a large S‐wave velocity jump at shallow depth (∼10–15 km depth). In addition, a negative arrival on the *k* = 0 harmonic at 5 s indicates a S‐wave velocity inversion around 40–50 km depth.

### 3.2 RF Back‐Azimuthal Harmonics Along a Trench‐Normal Profile

**Figure 3:**
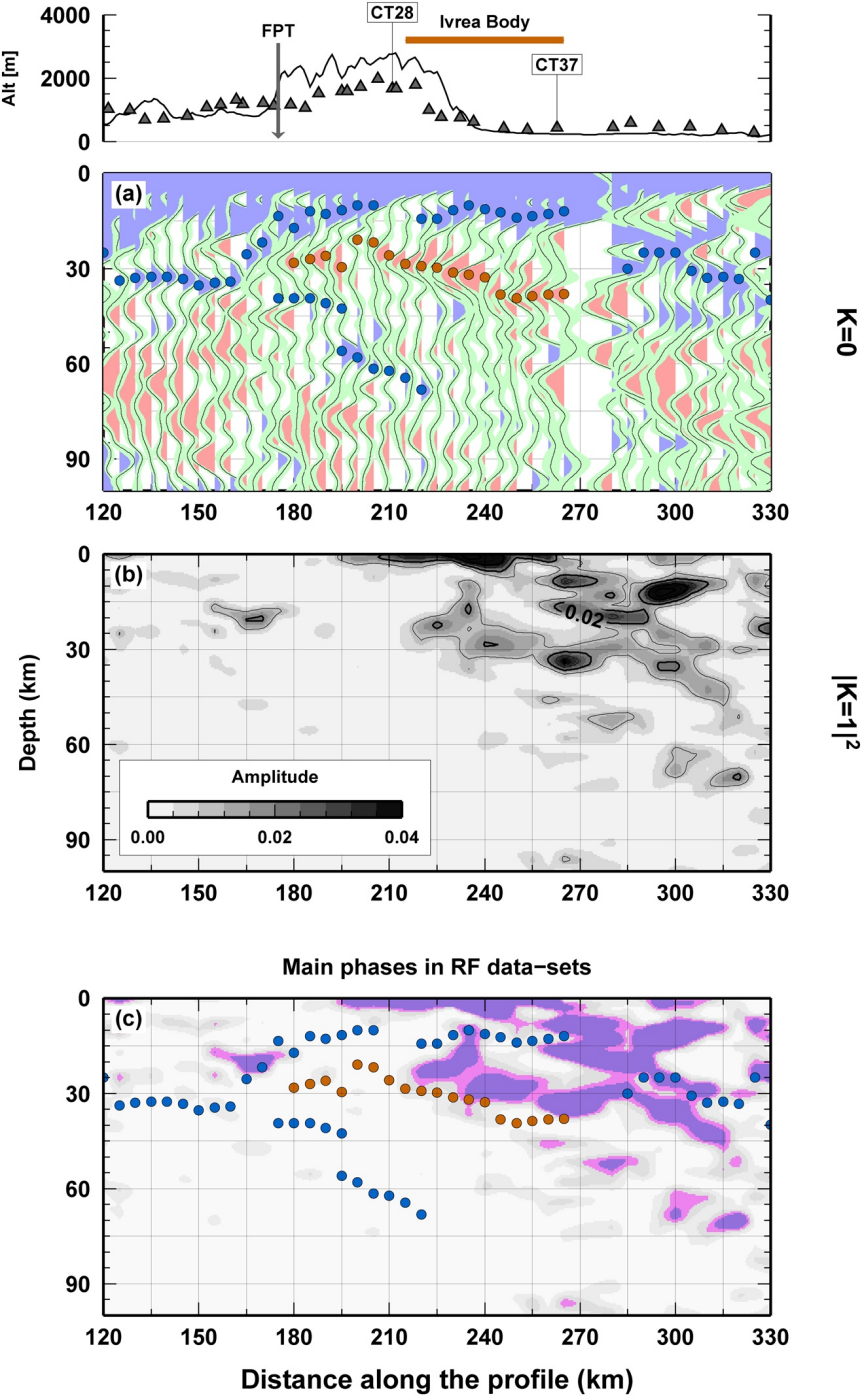
Angular harmonic decomposition of the receiver functions (RF) data set along profile AB (shown in Figure 1). Results are shown for the part of the profile between 120 and 330 km, across the Ivrea body (IB) (red line in the topography panel). The P‐RF and angular harmonics results for the entire profile are shown in (Figures S1 and S2). (a) Isotropic component (*k* = 0). Blue (positive) and red (negative) dots highlight the most important structures (i.e., seismic velocity discontinuities); (b) energy of the *k* = 1 (anisotropic) component. Darkest regions display highly anisotropic “interfaces," that is, the contact between anisotropic and isotropic bodies; (c) synoptic view of isotropic structures and anisotropic contacts as a summary of (a) and (b).

To appreciate the continuity of the features highlighted in the harmonics of the RF at station CT36 and, thus, the continuity of the main isotropic and anisotropic features of the IB, we computed the angular harmonics of P receiver functions along the entire CIFALPS profile following the approach depicted in Bianchi et al. (2010). To keep focusing on the scope of the study, in Figure 3 we display the results for the stations located around the positive gravity anomaly (*X* = 120 to *X* = 330 km) with particular attention to the ones located above it (*X* = 210 to *X* = 270 km). The entire profile is however presented in Figures S1 and S2.

Moving along the profile toward the NE, we observe that stations located outside the IB share similar isotropic interfaces (Figures 3a and S1). West of the FPT (i.e., from *X* = 120 to *X* = 150 km in Figure 3) and east of station CT37 (i.e., from *X* = 280 to *X* = 330 km), a continuous positive pulse is observed at a depth of ∼30 km, where Zhao et al. (2015) located the European and Adriatic Moho respectively. The pulse is less clear beneath the Po plain where the Moho has a very complex RF response due to multiples in the thick sediments. Both these interfaces are already imaged by several techniques and all agreed in location and extension as summarized in Figure 5b of Malusà et al. (2021). Conversely, in the region where the IB is supposed to extend (from *X* = 180 to *X* = 270 km, Figure 3), two continuous and positive pulses indicate shallower (∼10 km) and deeper (up to 70 km) dipping interfaces. The latter one is in continuity with the dipping European Moho and its depth and extension are again in agreement with the results of previous works (Beller et al., 2018; Lu et al., 2018; Solarino et al., 2018; Zhao et al., 2015, 2020). A negative (red) continuous pulse is observed at intermediate depth (20–40 km). The shallow positive arrival and the negative one confirm the presence of an anomalous high velocity body at shallow depth, as already found by the previous RF analysis on the same CIFALPS data set obtained by Zhao et al. (2015). This anomalous high velocity body is given by the IB, overlayed by the Dora Maira massif and it is where we identify the proto‐continental Moho discussed in the following sections.

In Figure 3c, we compare the results of the *k* = 0 and *k* = 1 harmonic analysis in the IB area. The most striking feature is the highly energetic arrival on the *k* = 1 harmonic in between the two (positive over negative) pulses on the *k* = 0 harmonic. This corresponds to another energetic arrival on the *k* = 1 harmonic near the surface (Figure 3c). Those two coupled energetic arrivals on the *k* = 1 harmonic define the top (at near‐surface depth) and the bottom (at ∼30 km depth) of a single highly anisotropic body which spans along the IB region documented by its gravity anomaly. Thus, the positive pulse on the *k* = 0 harmonic at ∼10 km depth, within the anisotropic volume, marks a large S‐wave velocity jump that defines our proto‐continental Moho. The negative pulse that indicates the velocity inversion arrives close to the bottom of the anisotropic body. It cannot be excluded that the bottom of the anisotropic body and the decrease in S‐wave velocity coincide. It is worth to note that the anisotropy in the easternmost part of Figure 3c is not related to the IB, because it is outside the area related to the high gravity anomaly (Figure 1), and its interpretation is out of the scope of this work.

Summarizing the observations made so far (Figure 3), we highlight: (a) the presence of highly anisotropic material in the area of the Ivrea Body between ∼5 and 50 km depth; (b) the coincidence between the location of the negative pulse and the bottom of the anisotropic volume; (c) the occurrence at all stations located above the IB, which confirms that teleseismic waves are sampling the same anomalous body; (d) the lateral continuity of such features, which suggests some kind of homogeneity in the seismic properties across the IB area.

### 3.3 Quantitative Estimation of Anisotropic Parameters by Monte Carlo Inversion

We quantify the anisotropic parameters in the IB through an investigation of the model space (NA, Sambridge, 1999). We define it for stations above the IB based on the pulses found in the RF data set (Figure 2) whose continuity has been documented along the profile (Figure 3). In particular, the continuity of the pulses depicted in Figure 3c prompt us to adopt a unique model space parameterization for all seismic stations (Table S1). For each station, the model includes six horizontal layers plus a half space representing the mantle. From top to bottom, the first layer represents the shallow crust, with thickness between 1 and 5 km; layers 2, 3, and 4 represent the anisotropic body, while the two remaining layers correspond to the isotropic deeper region. The last two layers are assumed to be isotropic, because we lack evidence for anisotropic material at depth greater than 40 km (no energy on *k* = 1 harmonic, Figure 3c). The S‐wave velocity is expected to increase between layers 2 and 3 to mimic the presence of a positive pulse in the RF data at 1–2 s, which we assume to reproduce the velocity jump at the top of the proto‐continental Moho. S‐wave velocity could in principle decrease at this interface because maximum velocity attributed to layer two is 3.5 km/s and minimum velocity in layer three is 3.0 km/s (Table S1). Following the indication of the *k* = 1 harmonic, layers 2–4 that correspond to the IB are considered as anisotropic. Minimum/maximum thickness of the anisotropic body is 20/55 km. Minimum/maximum values for the anisotropic parameters, that is, intensity and orientation of the symmetry axis, have a broad range of possible values with the intensity that is realistic for an assemblage of micaschists (Schijns et al., 2012) or serpentinized peridotite (Reynard, 2013). The choice of these lithologies, which have previously proposed for the IB (Zhao et al., 2020), implies the existence of “negative” anisotropy at depth, where the term “negative” means that the seismic velocity along the symmetry axis of the system is lower than the velocity along the plane normal to such axis. The choice of a “negative” anisotropy is made “a priori” and it is somehow subjective. As a matter of fact, Receiver Functions are unable to distinguish between positive and negative anisotropy, as found at first time by Sherrington et al. (2004) and clearly illustrated in Bianchi et al. (2008). RF patterns generated by a layer with “positive” anisotropy can be exactly reproduced by a layer of “negative” anisotropy, if the symmetry axis direction is rotated by 90° along its vertical plane (i.e., trend directions are opposite and plunge angles are complementary, see Bianchi et al., 2008). In Appendix A we illustrate the effect of “positive” anisotropy on the inversion results. The symmetry axis is free to rotate in 3D. The total number of investigated parameters is 23. Parameter space boundaries (i.e., min/max values for each parameter) are reported in Table S1. For each inversion, we perform a NA search on 21.000 models. Each inversion is independent from the others, thus coherent parameters between inversion results at different stations can be attributed to the presence of the IB and its physical properties.

**Figure 4:**
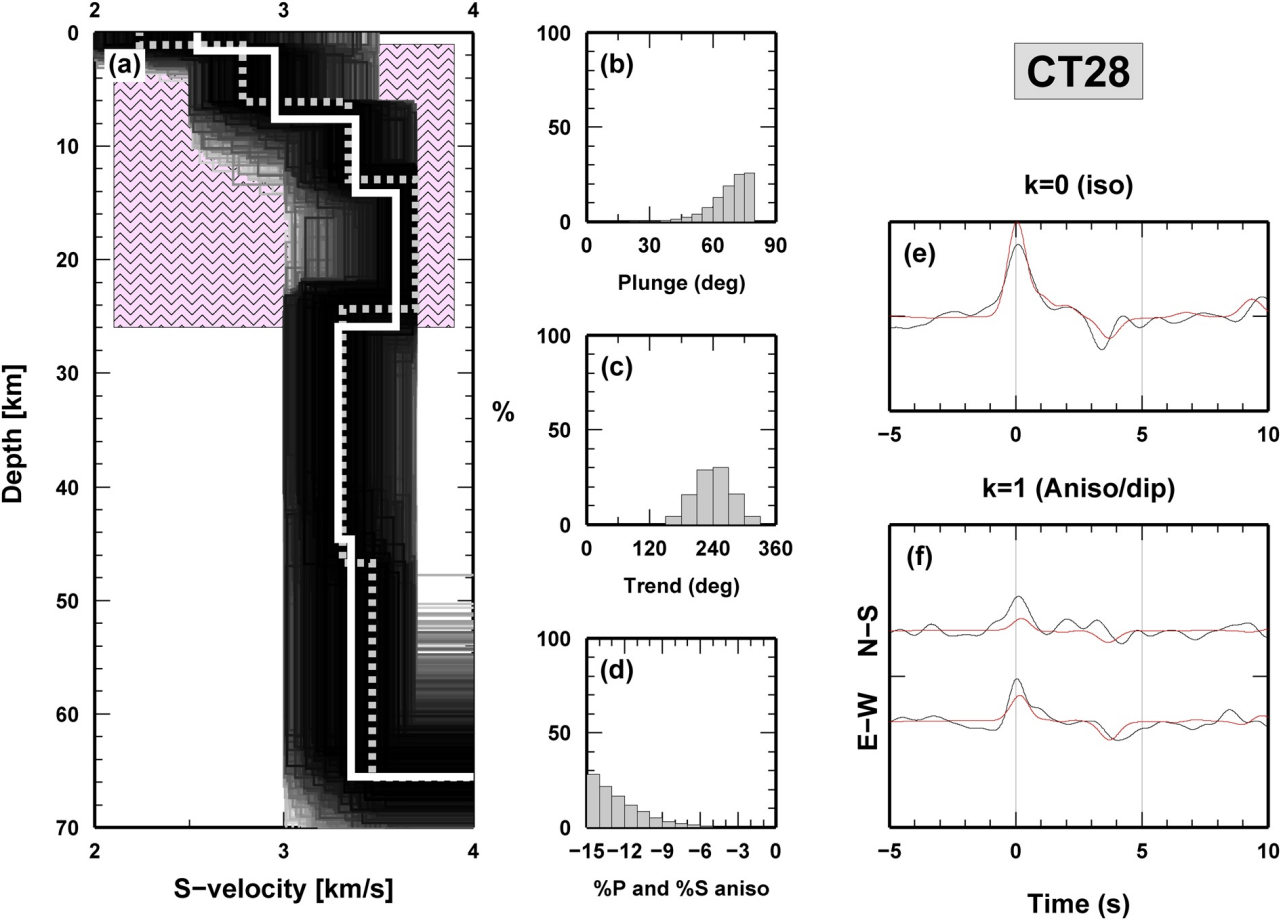
Example of results obtained solving the receiver functions (RF) inverse problem for S‐velocity and anisotropic parameters (trend, plunge and % of anisotropy) beneath station CT28 that is representative of stations above the Ivrea body (IB). S‐velocity profiles are shown in panel (a), where the dashed line displays the best‐fit model and the continuous thick white line is the best‐fit family mean model. The best S‐velocity model shows an almost continuous Vs increase from the surface to 15 km of depth (Vs = 3.7–3.8 km/s) before it decreases again below the IB (i.e., outside the pink textured area representing the IB). Panels (b)–(d): histograms of the anisotropic parameters for models in the best‐fit family. All parameters are well defined except intensity, which could be even higher than explored by the model space. Angular harmonics (observed in black and modeled in red, in panels e and f) computed using the best‐fit model, to illustrate the fit between synthetics and observations.

The NA inversion was performed for all stations located above the IB along the main profile (from CT28 to CT37, excluding CT30 because of scarcity of data) and also for those located off the transect, but located above the positive gravity anomaly (CT50 to the South, CT54 and CT55 to the North, Figure 1). For comparison, we also ran the NA inversion for two stations outside the IB area, CT20 and CT42, that in Figure 3 are located near the FPT (*X* = 180 km) and in the Po‐Plain (*X* = 315 km) respectively. An example of the results of the inversion is shown in Figure 4 for station CT28; the global search in the parameter space allows to invert for layer thickness, trend, plunge, and percentage of anisotropy and S‐velocity profile with depth. The quality of the inversion is assessed by comparing observed (black lines in panels e and f) and modeled (red lines in panels e and f) waveforms of the radial and transverse RF and the harmonic components, and by the distribution of the anisotropic parameters. For station CT28, the trend of the fast axis is well defined at 210°–270° from North, with a plunge of 75–80° from horizontal and a very strong anisotropy (−15%; Figures 4b–4d). The best S‐wave velocity model (dashed white line in Figure 4a) shows a continuously increasing Vs from the surface to ∼15 km depth up to 3.4–3.5 km/s with multiple velocity jumps. S‐wave velocity does not change between 15 and 26 km depth, then decreases in correspondence with the bottom of the anisotropic domain, assumed in the analysis as the results of the sum of thickness for layers 2, 3, and 4 (pink box in Figure 4a).

**Figure 5:**
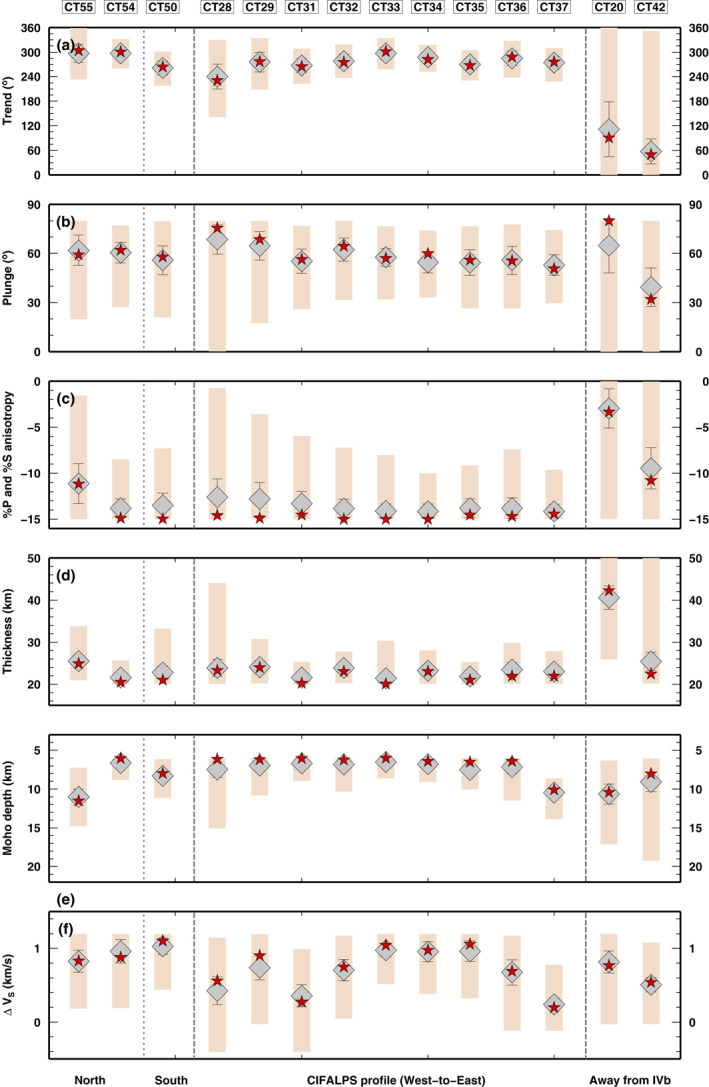
Values of parameters obtained by inverting for seismic and anisotropy parameters at stations located above the Ivrea body (IB) along the profile (CT28–CT37) and at stations to the north and south of it (CT50, CT54, and CT55). Stations CT20 and CT42 are located away from the IB and are inverted for comparison. From top to bottom, we report the obtained trend, plunge and % of anisotropy and thickness of anisotropic layer (panels a to d); the depth of the IB Moho (boundary between layers 2 and three in our model) and the S‐velocity variation across it (panels e to f). The results of the inversions are plotted as a single best solution (red star), mean value in the best‐fit family (gray diamond), and the min/max values found in the best‐fit family (colored bars).

Parameter values at station CT28 are related to the presence of the IB. We get similar results at all stations inside the area of Ivrea positive gravity anomaly (Figure S3). The inversion results are plotted in Figure 5 as a single best solution (red star), best‐fit family mean value (gray diamond) and distribution of values within the best‐fit family (colored bars). Stations located on Tethyan oceanic rocks and in the Dora‐Maira massif (from CT28 to CT33), and stations in the Po Plain (from CT34 to CT37) display a quite homogeneous anisotropic direction of 270° (from 240° at CT28 to 300° at CT33), a plunge ranging from 70° (for western stations) to 50° (for eastern stations) and an average percentage of anisotropy across all these stations about −14% ±1%. South of the main profile, CT50 has the same trend (240°), a plunge of 55° and the same percentage of anisotropy. Stations located north (CT54 and CT55) have an anisotropic direction closer to 300°, a plunge of 60° and different amounts of anisotropy, less than −15% for CT54 and ∼–10% for CT55. For all stations, the thickness of the anisotropic layer varies from 20 to 26 km, with a good agreement between best solution, mean value and the whole distribution. The proto‐Moho depth for most of the IB stations is shallower than 10 km, excluding stations CT37 and CT55, where it is deeper. The ∆Vs (i.e., the velocity jump at Moho interface) varies from 0.4 to 1.0 km/s with quite large differences between closer stations, again with an important uncertainty.

All these values are characteristic of stations located above the IB. Moving away from the positive gravity anomaly zone, the anisotropic properties obtained with the NA inversion are very different (e.g., CT20 and CT42, Figure 5), because the RF waveforms do not display any of the key features used to define the searched parameter space. All parameters recovered for CT20 and CT42, in particular the anisotropy parameters, have a scattered distribution as testified by the large min/max intervals (colored bars in Figure 5). This scatter means that inversion parameters are not constrained by the RF waveforms and results are not reliable, because the velocity model which applies elsewhere here does not (Figure S3).

## 4 Origin and Deformation of the Continental Moho

### 4.1 Rock Assemblage at the Continental Moho: Insights From Anisotropic Parameters

The results illustrated so far robustly indicate the presence at shallow depth of a rock volume with relatively high seismic velocity and rather homogeneous seismic properties in terms of bulk seismic velocity and anisotropic parameters (Figure 5). The rocks above and in the IB have a strong anisotropy of −14%, a symmetry axis trending 240°–300° with a plunge of 50°–70°. These best‐fitting values can be compared to published values of anisotropy measured on rock samples to infer the lithology and fabric of the IB and therefore, the lithology and fabric across the local proto‐continental Moho.

Intensity of anisotropy is strong for all stations along the transect (CT28–CT37), with average values up to −14% and minimum acceptable values of −10%. The same range of values is found for offline stations to the south (CT50) and north (CT54) of the main profile, except CT55, where significantly lower values are computed (−10%). Various studies investigated the anisotropy of rock samples from the Ivrea‐Verbano zone, representative of the mid‐ and lower crust. Khazanehdari et al. (2000) have shown that the percentage of anisotropy decreases with depth of burial, roughly following the presence of metasedimentary rocks, that in the “Serie dei Laghi” succession reaches ∼15%. In the same region, Barruol and Kern (1996) showed that felsic rocks with biotite and amphibole LPO (lattice‐preferred orientation) have the highest Vp‐anisotropy (10%). Our results show the same large intensity of anisotropy above and beneath the proto‐continental Moho, from the surface to the bottom of the IB. We can rule out the presence of a dry olivine assemblage because such rocks have less than 5% of anisotropy even though a single olivine crystal is highly anisotropic (Vp 24%, Vs 18%, Mainprice, 2015). Moreover, late stages of lower crust metamorphism (hydrated eclogite) would also be associated with low anisotropy (<4%, Christensen & Mooney, 1995) and thus excluded by our data. Pistone et al. (2020), using geophysical and petrological data to model the IB in the Valsesia area, proposed a lower crust composed by amphibole gabbros in the first 18 km depth and pyroxene hornblendites at greater depth, rocks characterized by low anisotropy. This hypothesis is based on the idea that the IB would have been involved in the formation of an igneous complex during magmatic underplating. The amount of anisotropy that we found in our inversion is not compatible with the rocks proposed by Pistone et al. (2020), so we should assume that the igneous complex these rocks would belong to, has a limited dimension, lower than the tens of km distant from CIFALPS stations.

Conversely, serpentinites are strongly anisotropic rocks associated with a subduction/collision environment that potentially exist in the depth range considered here, and that reach anisotropy higher than 20% when waves propagate through the foliation plane (Bezacier et al., 2010). A single block of serpentinite is unlikely due to the gravitational instability of serpentine rocks once formed. However, slices of serpentinized mantle may be assumed to accumulate and compose the high‐velocity IB. They can have variable degrees of serpentinization and, thus variable seismic velocity and anisotropic intensity depending on the hydration process and on the amount of fluids released from the lower crustal metamorphism or from the mantle wedge (Zhao et al., 2020).

The trend direction of the anisotropy found for stations across the gravity anomaly is similar to the fast velocity axis of azimuthal anisotropy calculated with the Pn phase by Díaz et al. (2013). Although their regional study has a much lower resolution than ours, they show that the fast direction of the Pn phase, which is refracted beneath the Moho, ranges between NE‐SW and E‐W directions at the boundary between Alps and Po Plain. In the same region and for the same stations as here, Salimbeni et al. (2018) analyzed SKS phases traveling through the upper mantle and found NW‐SE or NNW‐SSE directions of fast axes, very different from orientations obtained by RF decomposition. Few additional SKS splitting measurements, confirming this difference are given also by Petrescu et al. (2020). At station CT50, south of the main transect, the anisotropy trend obtained by RF inversion is similar to the ENE‐WSW Pn direction and again very different from the SKS measurements, that give an almost NW‐SE direction. The 280° fast axis orientation found at stations CT54 and CT55, located north of the CIFALPS transect, is instead in disagreement with the NE‐SW direction found both by Pn and by SKS results. The E‐W to NE‐SW directions are however in agreement with the regional fabric found by Barruol and Kern (1996) analyzing outcropping rock samples in laboratory experiments. In general we can then assume that for this region mantle (SKS) and shallower anisotropy (detected by Pn and RF) are different, and consequently also their origin. No previous work has measured the plunge direction of the fast‐velocity axis. We observe a gradual decrease of the plunge from west to east from ∼70° at CT28 to 50° at CT37. Comparable values are found for station CT50 (to the south) and lower values to the north (CT54 and CT55). Considering the trade‐off between type of anisotropy as explained in Appendix A, the plunge axis directions are remarkably in agreement with the directions which characterize the main tectonic structures of the study region, that is, all dipping toward the inner part of the Western Alps arc, with a tendency to flatten toward the east (Malusà et al., 2021). We suggest that such distribution could be related to different stages of deformation along the collisional front, reaching the maximum deformation in correspondence of CT31 located at the top of the Dora Maira dome, where the IB intruded at shallower depth, and near the highest values of Bouguer anomaly, that is close to CT34 station.

Summarizing, our lithologic model of the rock assemblage across the continental Moho comprises: an upper section of mid‐grade metamorphic rocks, with relevant schistosity, and a lower section composed of slices of highly serpentinized mantle rocks. Such highly deformed metamorphic rocks should have been exhumed/hydrated during subsequent stages of the orogen evolution, likely under different tectonic regimes but sharing the same main deformation direction. Variable amounts of serpentinization is foreseen (Solarino et al., 2018; Zhao et al., 2020), even if not robustly detected by our data. It is worth pointing out that the similarity of the RF data across all stations from CT28 to CT37 robustly supports the existence of such lithologic models all across the positive gravity anomaly (i.e., the IB).

### 4.2 Origin and Deformation Behavior of the Continental Moho

**Figure 6:**
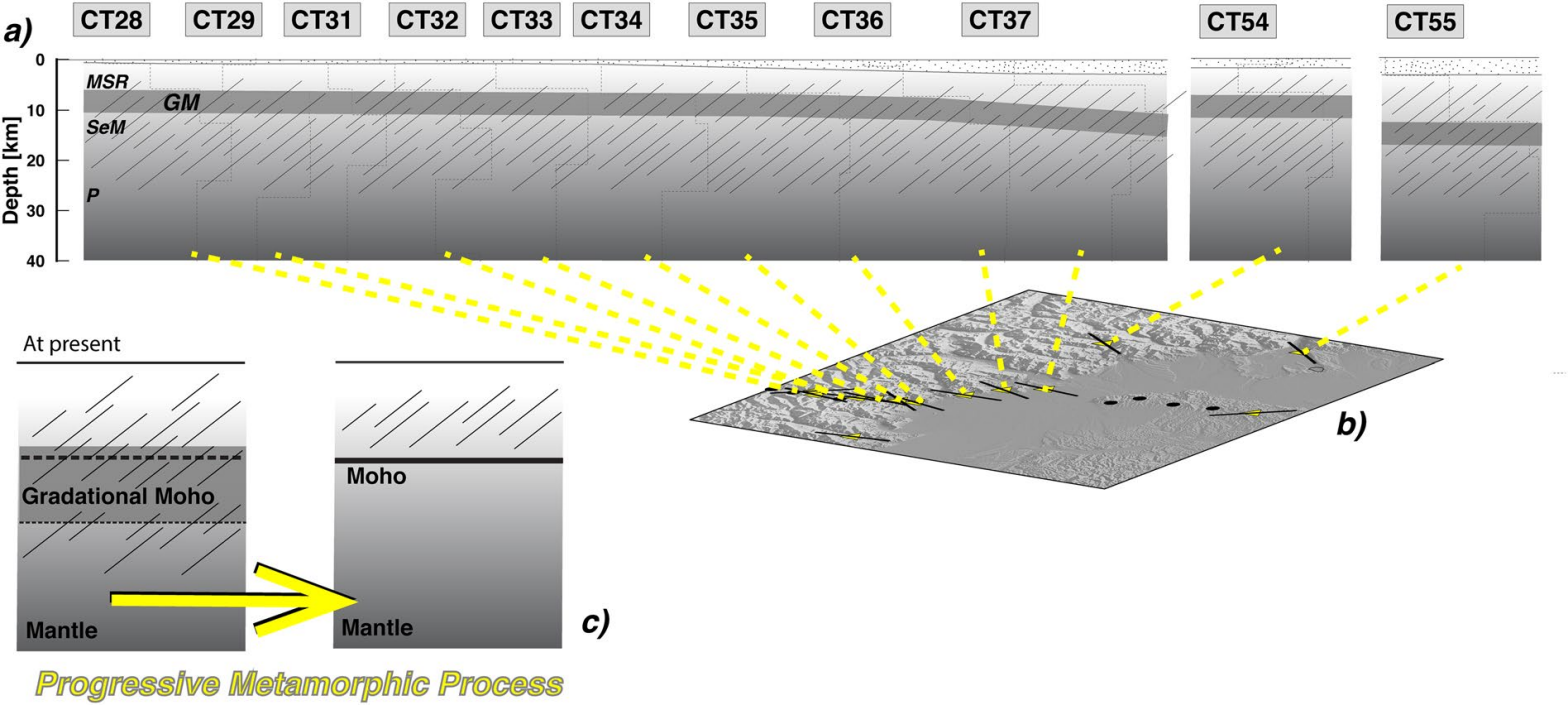
(a) Section drawn following velocity profiles (dotted lines, see Figure S3) for stations along the CIFALPS transect part and two stations located northeastward, all above the IB. Below the sedimentary cover (dotted pattern, 0 to 2–5 km), MSR, metasedimentary rocks; GM, Gradational Moho; SeM, Sliced serpentinized mantle; P, peridotite; referring to velocity profiles obtained in this work (dotted lines). The oblique pattern mimics the anisotropy distribution; (b) map of CIFALPS stations (yellow triangles) with black sticks drawn in the direction of the anisotropy trend; (c) on the left, cartoon illustrating a model similar to the structure detected in this work, proposed for the formation of a new continental Moho along a metamorphic/metasomatic front (modified by Eaton, 2006); on the right, the possible future structure after a progressing metamorphic process.

There are four main hypotheses for the formation of the continental Moho (Eaton, 2006): inherited old oceanic Moho, magmatic underplating, metamorphic/metasomatic event and regional detachment. Such hypotheses are not self‐exclusive and could co‐exist in different areas and in the same tectonic region. Our results, HP metamorphic rocks above serpentinized peridotite, can be somehow generalized and connected to the different hypotheses (Figure 6). At a first glance, the presence of serpentinized mantle rocks just below the Moho would pose the origin of our Moho close to the first hypothesis, but the episodes of exhumation of the HP rocks of the Dora Maira dome should have created a visible (not transparent) local Moho (see Figure 3 in Eaton, 2006).

Even if the process generating the metamorphism in the lower crust is different from what postulated by Eaton (2006), our conceptual model shares some similarity to the model that considers a metasomatic/metamorphic origin for the Moho (Figure 6). In such a model, the action of the metamorphic process should produce a phase transformation that changes the elasticity of the rocks near the crust‐mantle boundary and likely their anisotropic properties. At the end of the process the Moho would appear as a “thick interface,” in which the velocity changes gradually producing difficulties on its determination with seismological tools. In fact, we observe that our models for the IB generally define two consecutive velocity increases at the Moho depth range at about 5–8 km and, given the frequency content of the teleseismic waves used in the analysis, we can't exclude a transitional boundary 2–3 km thick at each interface. Thus, we suggest that a single, sharp interface (and easily spottable?) is unlikely to represent the continental Moho we detected in this study. The velocity jump in our case is also partially “obscured” by the presence of relatively low‐velocity materials (i.e., serpentinized peridotite) below the proto‐continental Moho (Figure 6). Recently Pistone et al. (2020), using gravity and seismological evidence, proposed magmatic underplating as a possible hypothesis for petrological reconstruction of the origin of the IB into the European lower crust. However, in our opinion, this hypothesis is not feasible in our area of interest at least for three reasons: (a) the Pistone et al. (2020) study points to a zone (Valsesia region) ∼100 km of distance from our study region; (b) signature of the supposed magmatic underplating is collected by outcropping Ivrea‐Verbano rocks that in our region are not present, cause we are located where IB is completely buried at depth; (c) the lithologies proposed by the authors (amphibole gabbros in the first 18 km followed by pyroxene hornblendites) are not compatible with the intensity of anisotropy we obtained from the NA inversion.

Our preferred model for the local formation of the continental Moho could also explain discordant reflectivity between lower crust and upper mantle, where the lower crust has laminar reflectivity, absent beneath the Moho (Eaton, 2006). Considering ours as a model of a young continental Moho, after some time, in case of preservation of the IB Moho, the mantle rocks beneath it are likely to undergo thermal relaxation which would not preserve serpentinization, slowly promoting antigorite breakdown and fluid release (Hacker et al., 2003), with two main consequences (Figure 6c). First, the velocity contrast at the Moho would dramatically increase due to the dehydration of the peridotite. Second, the anisotropic behavior of the serpentinite rocks found here would be not preserved at all during antigorite breakdown (Chollet et al., 2011). Conversely, the lower crust would not reach critical PT conditions promoting final eclogitization of the (former) HP rocks and, thus, anisotropic (laminar) characteristics would be locally preserved. Finally, given the possibility of antigorite breakdown during the final stages of Moho maturation, and consequent disruption of the anisotropic behavior in the serpentinized mantle, we can anticipate the absence of a similar reflectivity (lamination) between lower crust and upper mantle for a mature continental Moho. In our case, such absence should not be considered as a signature of different deformation regimes or decoupling of the deformation front at the Moho and, thus, potentially promoting the delamination process in favorable tectonic settings (as postulated in Eaton, 2006). In our model, the anisotropic parameters above and below the Moho display consistently the same values, and could have been oriented within similar stress conditions. But, as described above, such anisotropic characteristics could not survive in the upper mantle for a long time, recasting our model into the picture described by Eaton (2006), that is, laminar lower crust and absence of coherent reflectivity in the upper mantle. We suggest that having different reflectivity between crust and upper mantle should be carefully investigated before considering it a factor promoting delamination.

## 5 Conclusions

We analyzed teleseismic receiver function across the Ivrea Body (Northern Italy) using the data set of the CIFALPS seismic network (Zhao et al., 2016), where the preservation of the crust‐mantle boundary at shallow depth allows to investigate in details both isotropic and anisotropic seismic properties at the lower‐crustal and upper‐mantle depth‐levels, and to give new insights into the origin of the continental Moho. The main points raised by the work are:

1. The Ivrea Body and overlying crustal rocks display a strong anisotropic behavior (−14% anisotropy) with well‐constrained trend and plunge. The Ivrea Body is a unique phenomenon that involves all the structures in three‐dimensional, with similar isotropic and anisotropic parameters.
2. Our observations are consistent with a metamorphic/metasomatized origin for the continental Moho, where the Moho is inherited by the pre‐orogenic plates and reworked during the orogen process.
3. Given the coherence and continuity in the anisotropic parameters between lower crust and upper mantle, we suggest that, at least in this case, the delamination of the two units is not favored (strong coupling).
4. Given the possibility of antigorite breakdown, and consequent disruption of the anisotropic behavior in the serpentinized mantle, we suggest that the absence of similar reflectivity (lamination) between lower crust and upper mantle should not be considered a factor favoring delamination (as postulated in Eaton, 2006).

## Conflict of Interest

The authors declare no conflicts of interest relevant to this study.

## Supporting information

Supporting Information S1

## Figures and Tables

**Figure A1:**
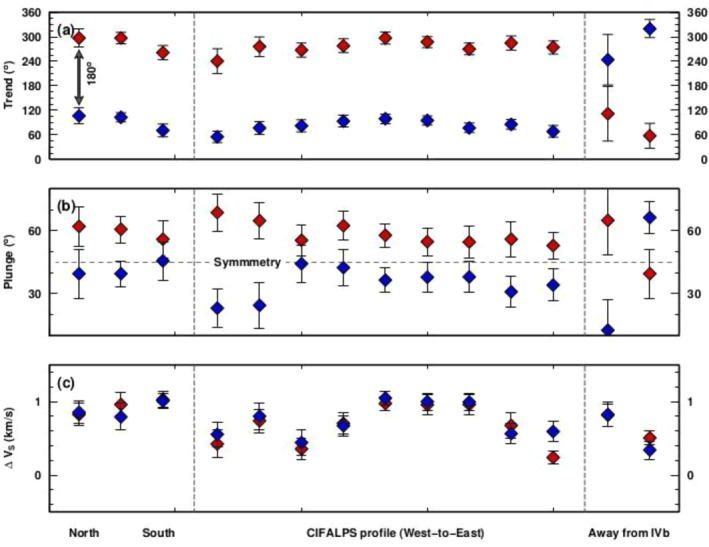
Comparison of the results obtained from NA inversion using “negative” (red) and “positive” (blu) anisotropy.

## Data Availability

All waveforms analyzed are freely accessible through EIDA portal (http://orfeus-eu.org/webdc3/) and details of the CIFALPS seismic network are provided in Zhao et al. (2016; https://doi.org/10.1029/2020GC009466).

## Appendix A Results Obtained Using “Positive” Anisotropy

### A.1

The simplified model that we adopt for seismic anisotropy, *hexagonal anisotropy with one single, freely‐orientable, symmetry axis*, can be declined in two families: “positive” anisotropy, also called “melon‐shape” anisotropy, where the seismic velocity is larger for a wave propagating in the direction of the symmetry axis with respect to the seismic velocity of a wave propagating along the normal plane; and “negative” anisotropy, also called “pumpkin‐shape” anisotropy, where the opposite situation is presented, that is, the seismic velocity for a wave propagating along the symmetry axis is smaller than for a wave propagating along the normal plane (Levin & Park, 1997; Sherrington et al., 2004).

It has been proven that the signal generated from a model of the two families, and recorded in a RF data set, can be exactly reproduced by one model in the other family (Sherrington et al., 2004; see also Bianchi et al., 2008; for synthetic examples). Basically, RF data can't purely discriminate between the two families. It is a widely used approach in RF inversions to decide “a‐priori” which family to be considered in the parameterization. Here, given the lithologies considered, we opt for a parameterization with a negative anisotropy. In this Appendix, we show the results obtained when we invert the data considering a parameterization with a positive anisotropy. As a rule of thumb, the two members of the two families which are able to reproduce the observed data at the same level should have: (a) opposite trends, that is, their trends differ by 180°; and (b) complementary plunges, that is, plunges can overlap if one is rotated 90° on the vertical plane defined by the trends direction. The second condition means that the plunge angles of the two members are symmetric around 45°.

In Figure A1, we illustrate our results. In panel (a) we report the trend direction for the two families, in terms of average values found in the two inversions for all stations. To clarify, the red symbols refer to the results presented in the main text (Figure 5). Blu symbols refer to the results obtained with a parameterizations which considers a positive anisotropy. As anticipated, the trend directions, in the results obtained with the two different parametrization, are almost perfectly spaced by 180°, for all stations deployed on the IB. The plunge axes are less constrained, but again, our results overall confirm the predictions. In fact, for each station deployed on the IB, plunge axes obtained with the two parameterizations are always one smaller and one larger than 45°. Finally, we can confirm that isotropic 1D Vs profile retrieved from the inversion is not strongly affected by the choice of the family used in the parametrization. For example, the velocity jump at the proto‐Moho (panel c) is almost the same for the two inversions, for each station deployed on the IB.
