# Supplementary material for: Insights Into the Origin and Deformation Style of the Continental Moho: A Case‐Study From the Western Alps (Italy)
Source: J Geophys Res Solid Earth. 2021 Jun 23;126(6):e2020JB021319. doi: 10.1029/2020JB021319 (PMC8365725; doi:10.1029/2020JB021319)
Supplement: Supplementary file 1 — Supporting Information S1 [file JGRB-126-e2020JB021319-s001.pdf]

Journal of Geophysical Research – Solid Earth

Supporting Information for

Insights into the origin and deformation style of the continental Moho: a case-study from the Western Alps (Italy)

Simone Salimbeni [a], Nicola Piana Agostinetti [b,c], Silvia Pondrelli [a], and CIFALPS Working Group*

[a] Istituto Nazionale di Geofisica e Vulcanologia, Bologna, Italy

[b] Department of Earth and Environmental Sciences, University of Milano Bicocca, Italy

[c] Department of Geodynamics and Sedimentology, University of Vienna, Austria

Contents of this file

Figure S1….page 2
Figure S2….page 3
Figure S3….pages 4,5,6
Figure S4….page 7
Table S1….page 8

Introduction

Supplementary material supporting the manuscript is composed of 4 Figures and 1 Table. In each page only 1 supporting material is displayed except for Figure S3 that is separated in 3 continuous pages.

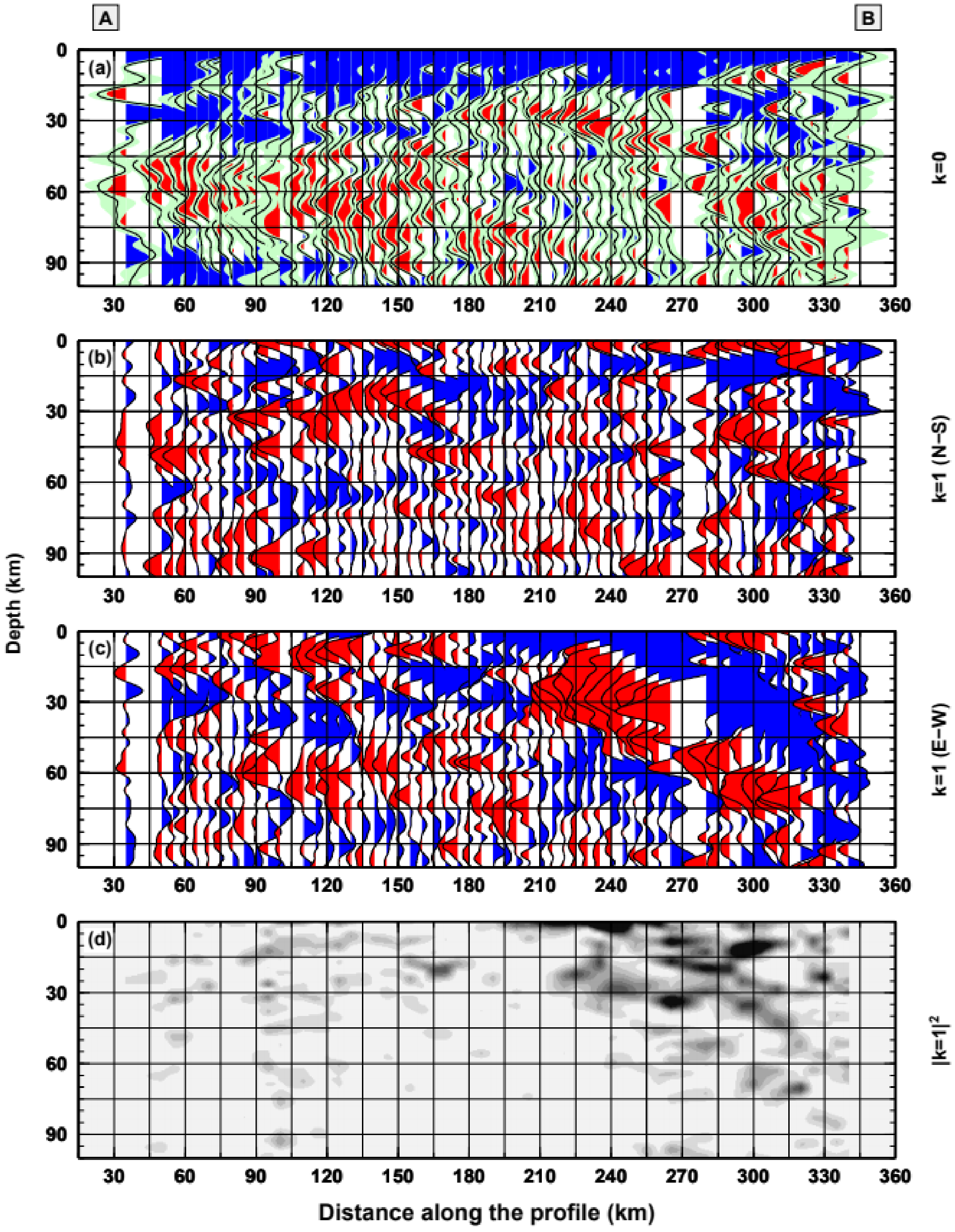

Figure S1. P-RF along the entire profile (see map in Figure 1)

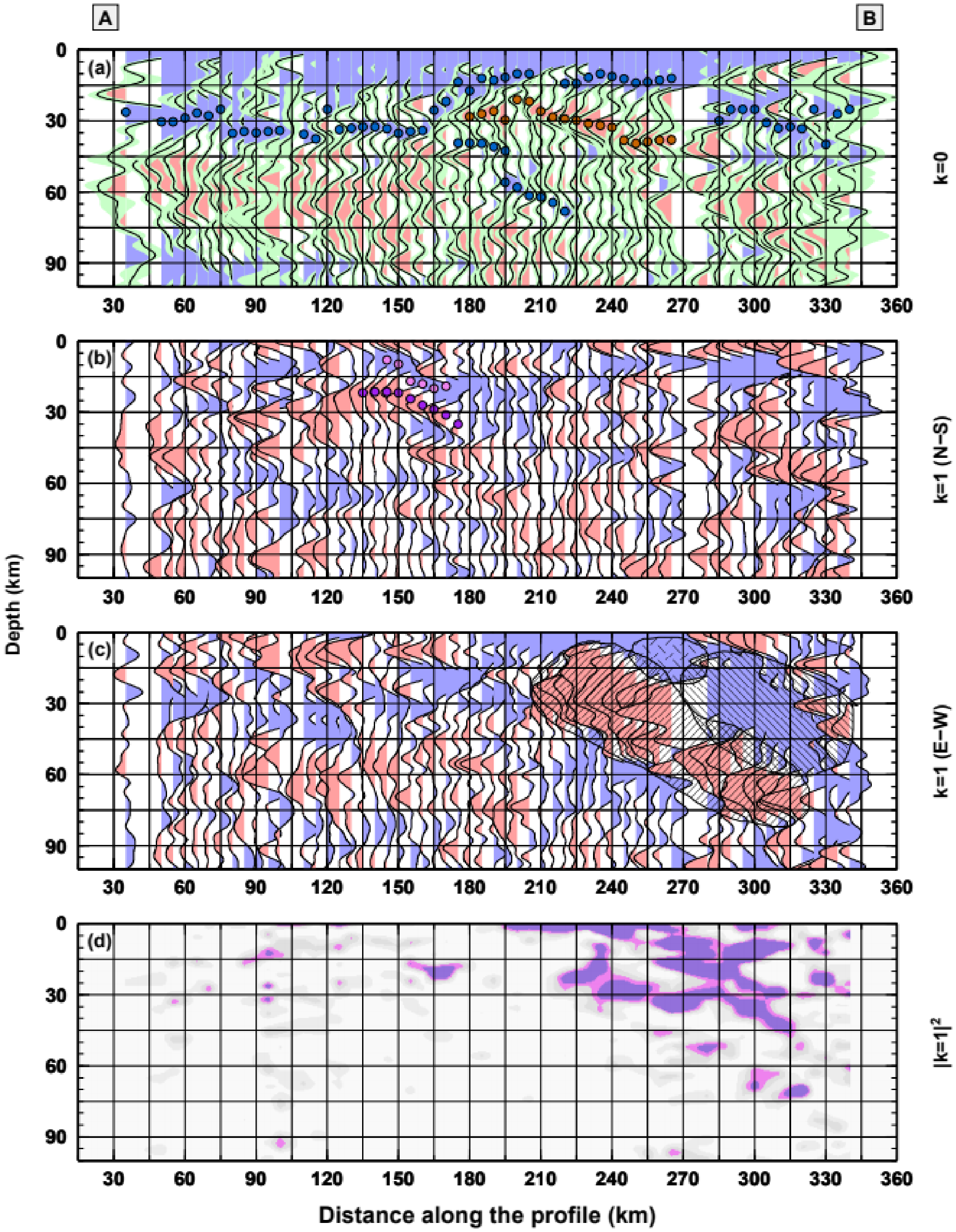

Figure S2. Angular harmonics results for the entire profile (see map in Figure 1)

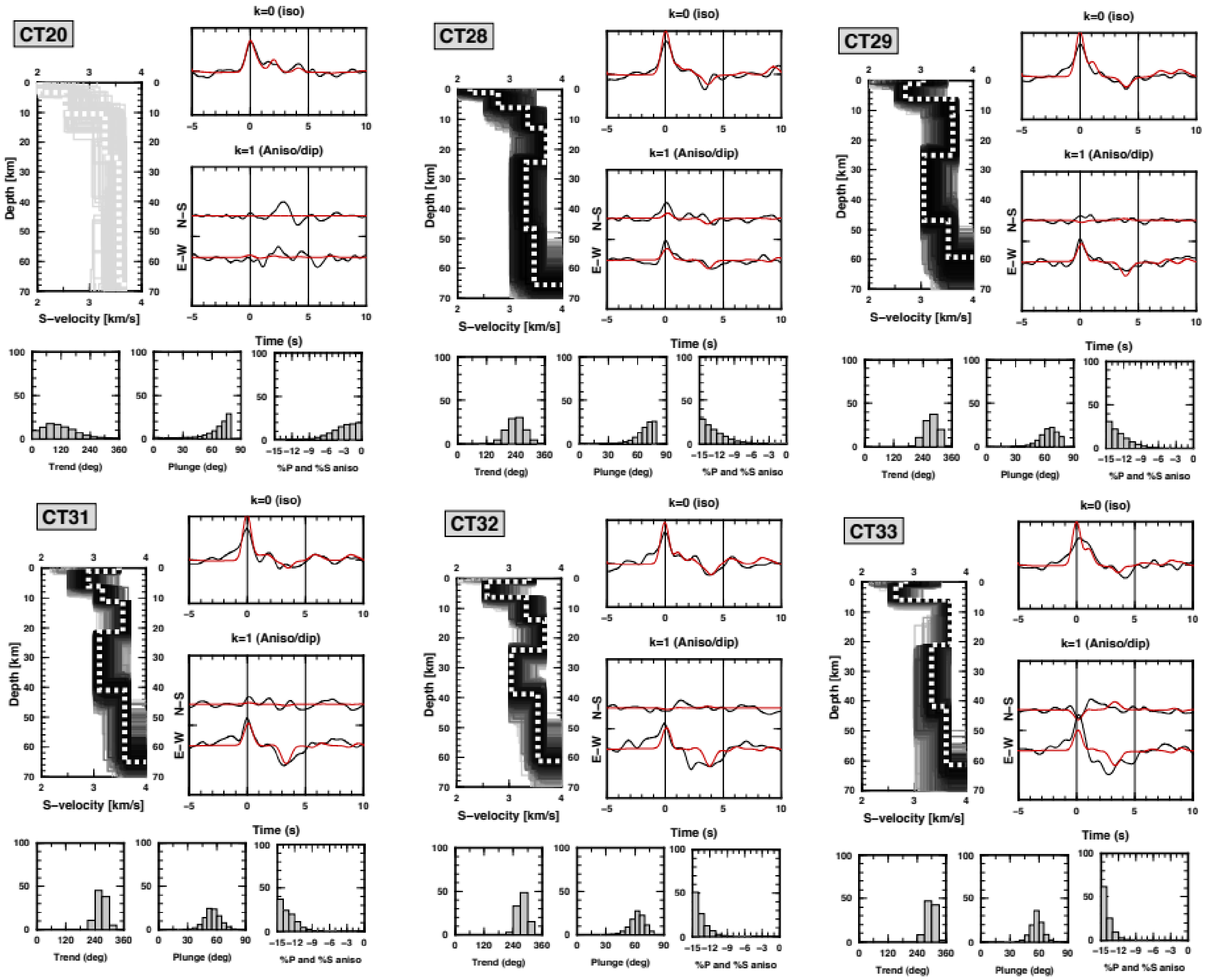

Figure S3. Results obtained solving the RF inverse problem for the profile of S-velocity and anisotropic parameters (trend, plunge and % of anisotropy) beneath all the stations analysed. Legend is the same of Figure 4. *Continue in the next page…*

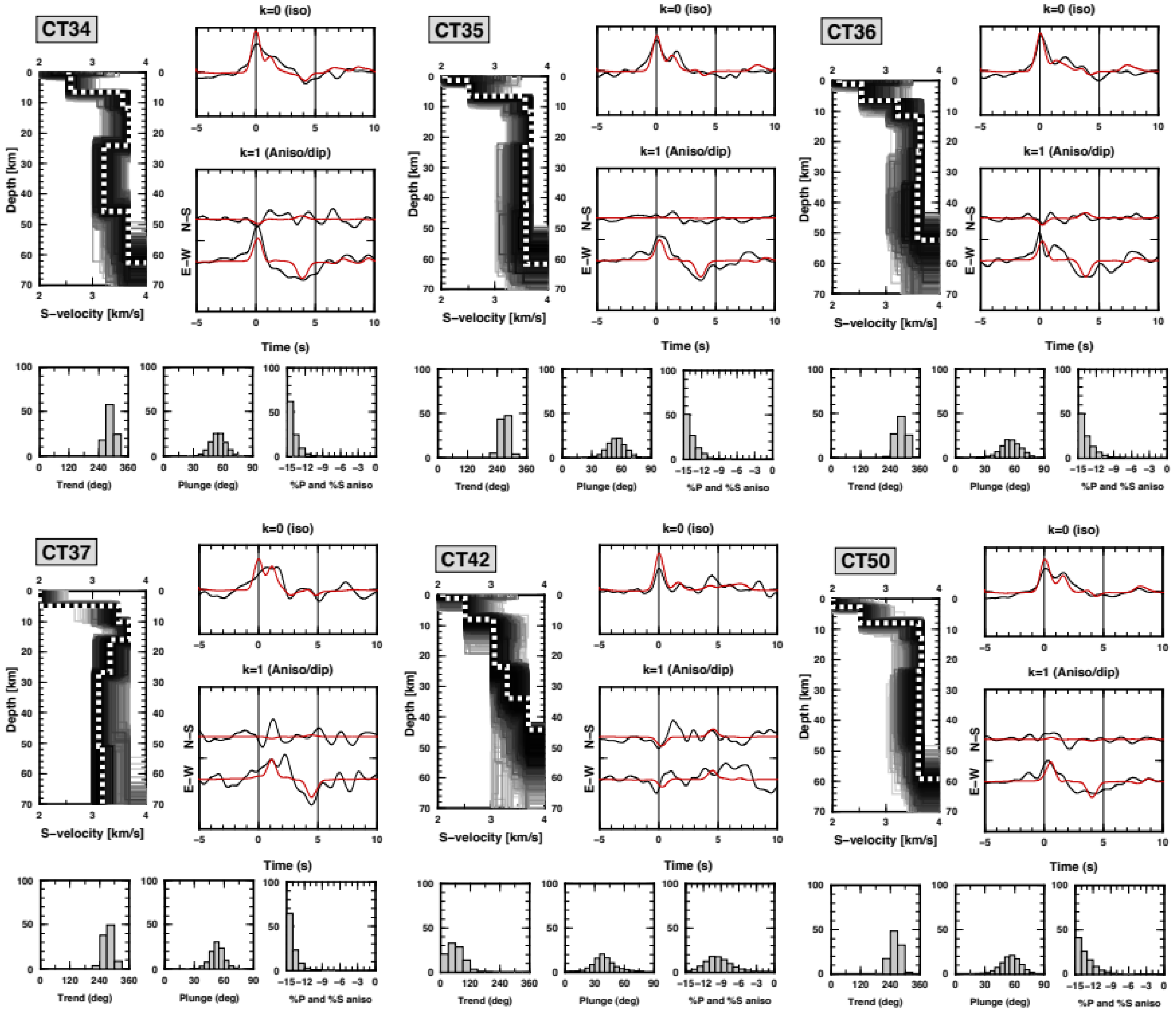

Figure S3. Results obtained solving the RF inverse problem for the profile of S-velocity and anisotropic parameters (trend, plunge and % of anisotropy) beneath all the stations analysed. Legend is the same of Figure 4. *Continue in the next page…*

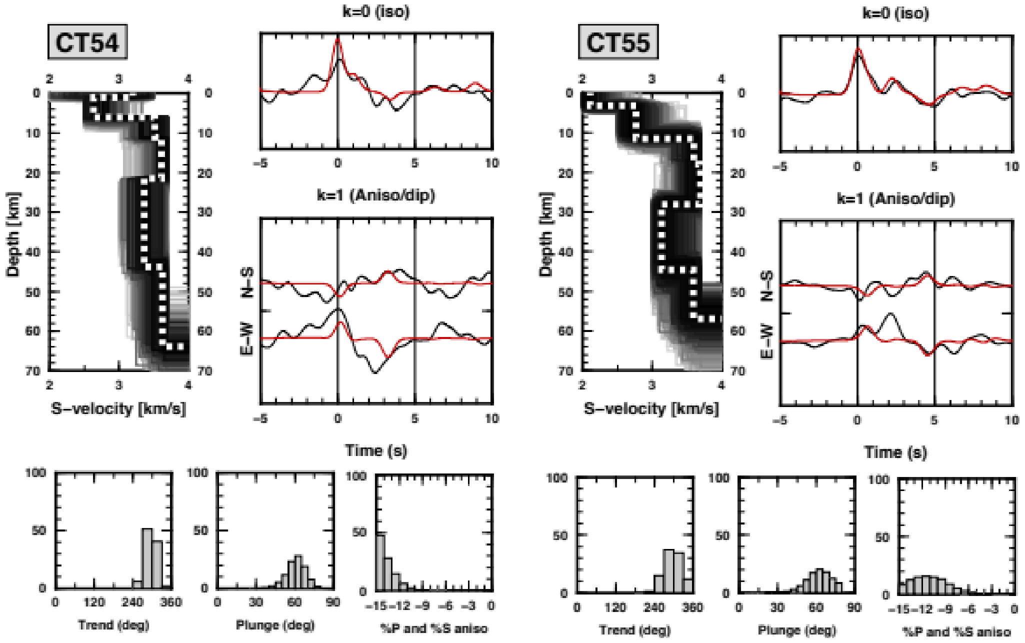

Figure S3. Results obtained solving the RF inverse problem for the profile of S-velocity and anisotropic parameters (trend, plunge and % of anisotropy) beneath all the stations analysed. Legend is the same of Figure 4.

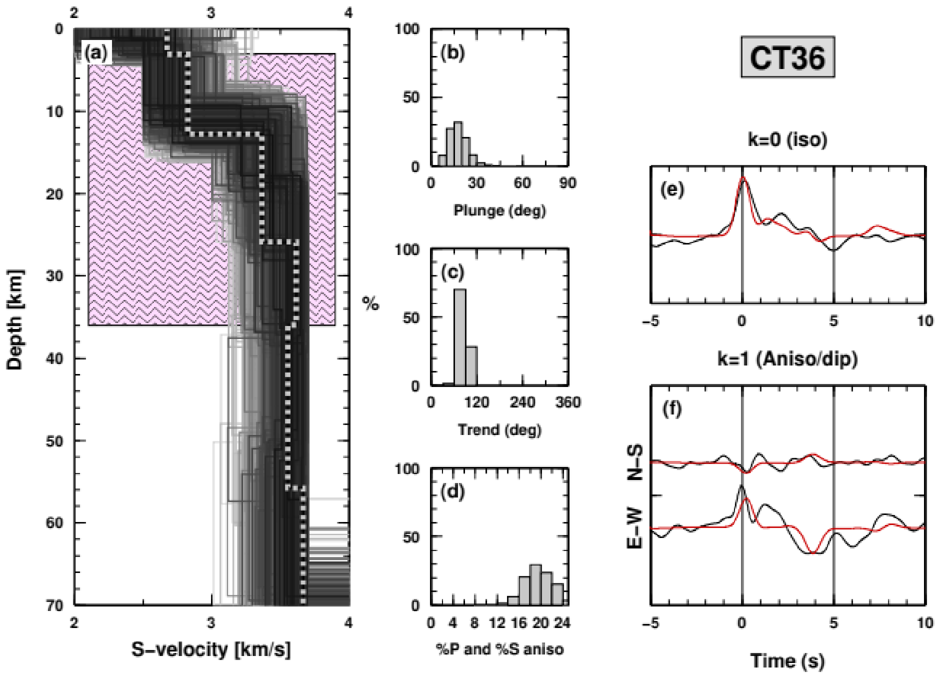

Figure S4. Example of results obtained solving the RF inverse problem for S-velocity, trend, plunge and a % of anisotropy ranging from 0-25% beneath station CT36. Legend is the same of Figure 4.

	Thick (km)	Vs (km/s)	VpVs	Anisotropy %P and %S	Trend (º)	Plunge (º)
**MINIMUM**						
	1	2,00	1,7			
	5	2,50	1,7	-15 (0)	0	0
	5	3,00	1,7	-15 (0)	0	0
	10	3,00	1,7	-15 **(0)**	0	0
	10	3,00	1,7			
	10	3,00	1,7			
	0	4,05	1,7			
**MAXIMUM**						
	5	3,50	1,9			
	15	3,50	1,9	0 (15)	360	80
	15	3,70	1,9	0 (15)	360	80
	25	3,70	1,9	0 (15)	360	80
	25	3,70	1,9			
	25	3,70	1,9			
	10	4,45	1,9			

Table S1. Space parameters variation used in the anisotropic properties calculation
